# Reply to the letter for the manuscript “The effect of proprioceptive vestibular rehabilitation on sensory-motor symptoms and quality of life”

**DOI:** 10.1055/s-0046-1817806

**Published:** 2026-04-29

**Authors:** Gülfem Ezgi Özaltın, Burcu Talu, Tuba Bayındır

**Affiliations:** 1 Inonu University, Faculty of Health Sciences, Physiotherapy and Rehabilitation Department, Malatya, Turkey.; 2 Yüksek İhtisas Unıversity, Medicalpark Ankara Hospital, Ear Nose and Throat Clinic, Ankara, Turkey.

Dear Editor,

Ventura et al. Thank you for your interest in our article titled “The effect of proprioceptive vestibular rehabilitation on sensory-motor symptoms and quality of life” and for your valuable feedback. I have carefully examined the Cohen's d and PS values you provided in your letter. However, in order to reflect the data we obtained within the scope of the study in a more reliable and consistent way, we have recalculated these values and presented them in a tabular form (Appendix 1).

The effect size (Cohen's d) value was obtained using the online calculator Psychometrica, and the Probability of Superiority (PS) value was calculated from this d value using the formula Φ (d/√2). 1 Prior to the initiation of the study, a power analysis was conducted with a significance level of α = 0.05 and a statistical power of 1-β = 0.80. Based on an anticipated average change of 27 points in the Dizziness Handicap Inventory (DHI) score, the analysis indicated that a minimum of 27 participants would be required, with at least 9 individuals per group. This minimum sample size was successfully achieved by the end of the study. 2 This calculation was made to minimize the probability of Type II error and the statistical power (1-β) was found to be sufficient.
